# Test-retest reliability of a functional electromechanical dynamometer on swing eccentric hamstring exercise measures in soccer players

**DOI:** 10.7717/peerj.11743

**Published:** 2021-07-14

**Authors:** Antonio Jesús Sánchez-Sánchez, Luis Javier Chirosa-Ríos, Ignacio Jesús Chirosa-Ríos, Agustín José García-Vega, Daniel Jerez-Mayorga

**Affiliations:** 1 Department Physical Education and Sports. Faculty of Sport Sciences, Universidad de Granada, Granada, Spain; 2 Faculty of Rehabilitation Sciences, Universidad Andres Bello, Santiago, Chile

**Keywords:** Isokinetic, Eccentric, Lower extremity, Dynamometer, Hamstring, Soccer players

## Abstract

**Background:**

The use of a functional electromechanical dynamometer (FEMD) has been proposed as a valid and effective tool to evaluate specific movement patterns. The aim of this study was to determine the reliability of FEMD on swing eccentric hamstring exercise (SEHE) measures in soccer players.

**Methods:**

Nineteen federated male soccer players (20.74 ± 4.04 years) performed the SEHE at three different isokinetic velocities (20–40–60 cm/s). These evaluations were conducted in four sessions, two for familiarization and two for registration. The average and maximum load (N) of the three isokinetic velocities was calculated from the values obtained from the FEMD (Dynasystem^®^, Bangalore).

**Results:**

The main results of this research showed that the reliability was high for the average load in the condition of 40 cm/s, presenting the highest ICC value (0.94). For maximum load, reliability was high in the condition of 20 cm/s. The manifestation of the most reliable load was the maximum load (ICC = 0.91–0.87).

**Conclusions:**

FEMD (Dynasystem^®^, Bangalore) is a reliable device to evaluate the eccentric strength of the hamstring muscles in soccer players.

## Introduction

The cause of hamstring injuries in soccer players is often multifactorial (Liu et al., 2012), in nature and is a persistent problem with a high economic cost for clubs (€500,000 per month), as they constitute more than a third of all lesions (12%), the most common subtype (Elliott et al., 2011; Ekstrand, Hägglund & Waldén, 2011; Ekstrand, Waldén & Hägglund, 2016; Jones et al., 2019). Imbalance of muscle strength with a low eccentric hamstring/concentric quadriceps ratio (Hecc:Qcon) is one of the three most common modifiable risk factors (Croisier et al., 2008; Fousekis et al., 2011; Green et al., 2020). For this reason, prevention and performance optimization programs (Morin et al., 2015; Mendiguchia et al., 2015) focus on modifying the eccentric hamstring strength deficit and the Hecc:Qcon strength ratio (Croisier et al., 2002; Opar et al., 2013b). Many injuries of the lower extremities can occur when the hamstrings do not generate equivalent counter torque to decelerate a high anterior tibial shear in extended knee movements, which are induced by quadriceps maximal torque (Ruas et al., 2019).

In this context, exercises such as the Nordic hamstring exercise (NHE) have emerged as one of the most widely used exercises for the prevention and optimization of the hamstring muscles, as it increases the eccentric strength of the hamstring and decreases the incidence of injury (Maniar et al., 2016; Al Attar et al., 2017; Vatovec, Kozinc & Šarabon, 2019) although it has several limitations at the functional level, from the anatomical (only focuses on the knee, completely bypassing the hip joint) and physiological (is very demanding) point of view (Bourne et al., 2018).

Another option to evaluate the different types of muscle contractions includes angular isokinetic dynamometers (AID). Which are recognized as the gold standard for evaluating the eccentric strength of the knee flexor muscles, but lack practical utility in comparison with other devices (Opar et al., 2013a; Van Dyk, Witvrouw & Bahr, 2018; Dvir & Muller, 2019). AIDs, like the NHE, do not have transference to the actual playing actions that occur during the injury mechanism in soccer players, as the movement is focused only on the knee joint while the hip remains static throughout the entire run (Bourne et al., 2018).

Therefore, it seems logical to perform transferable exercises such as the swing eccentric hamstring exercise (SEHE). This exercise, we propose, simulates the late swing phase of high speed running by combining hip flexion with knee extension, a mechanism performed during the sprint (Chumanov, Heiderscheit & Thelen, 2011; Chumanov et al., 2012). Similarly, it is also important to use measuring devices that allow movements similar to sports specific movement to be replicated, such as a functional electromechanical dynamometer (FEMD), which allows us to assemble the eccentric phase from the concentric phase and execute the movement at different contraction velocities (Jerez-Mayorga et al., 2019, 2020; Rodriguez-Perea et al., 2019, 2021; Soriano-Maldonado et al., 2019; Fàbrica et al., 2020; Martinez-Garcia et al., 2020; Machado-Payer et al., 2020) . According to Dvir & Muller (2019) multiarticular isokinetic dynamometers (MID) such as FEMD can be applied validly and effectively to evaluate specific movement patterns, since there is no situation or action where only one muscle is working in isolation.

Therefore, the aim of this study was to determine the reliability of a FEMD on SEHE measures in soccer players. We hypothesized that (I) this new test will be a reliable method for the assessment of eccentric strength in hamstring, and (II) maximum load would be more reliable than average load. These results could be of great importance when carrying out prevention and optimization programs for the hamstring muscles in soccer players, as they would work on the same gesture of the injury mechanism.

## Materials & methods

### Design

One week before the beginning of the experimental phase, all the participants were subjected to two familiarization sessions to learn the correct technical execution of the procedure to be performed through the functional performance of knee flexion and extension actions using different isokinetic velocities and muscle contractions (concentric and eccentric). A FEMD in its isokinetic mode was used to evaluate the reliability of the SEHE (Dynasystem, Symotech, Granada, Spain) (Rodriguez-Perea et al., 2021). These evaluations were carried out in four sessions, two for familiarization and two for registration at three different isokinetic velocities (20–40–60 cm/s), requiring a maximum effort during each of the repetitions. To avoid any signs of fatigue, a week was left between each data logging session.

### Participants

Nineteen federated male soccer players (*n* = 19, age = 21 ± 4.04 years, height = 177.00 ± 5.41 cm, weight = 73.35 ± 9.00 kg, BMI = 24 ± 2.41 kg/m^2^ and playing experience = 11.37 ± 3.00 years) voluntarily participated in this study. The inclusion criteria were (I) having at least five years of consecutive competitive experience before measurements; and (II) no previous hip, knee, or thigh injuries in the last six months. Exclusion criteria were (I) participation in any additional strength training program during the weeks the study was conducted; and (II) not attending one or more assessment sessions during the entire data collection process. All participants were invited to maintain their normal levels of physical-sporting activity throughout the exploratory process, although they were advised to avoid strenuous practices during the 24 h before each evaluation session. The players trained three times a week for 90 min and played an official match at the weekend.

All subjects were informed of the potential risks associated with the experimental procedures before giving written informed consent to participate. The study conformed to the ethical standards set by the Declaration of Helsinki. Ethics approval was granted by the Human Research Ethics Committee of the University of Granada, Spain (IRB approval: 822/CEIH/2019).

### Procedures

The isokinetic tests were evaluated after an aerobic warm-up protocol and posterior chain exercises. The aerobic warm-up consisted of 5 min on a cycle ergometer, at a resistance of 100 watts (W) and with a cadence of 70–80 r/min. Subsequently, three exercises were carried out to mobilize and activate the hamstring and gluteal muscles (10 repetitions with each leg): hip extension, unilateral bridge, and swing phase of high-speed running with elastic band in the ankle. Once the warm-up was completed, the subject performed a series of five approach repetitions on the FEMD at a constant isokinetic velocity in the concentric and eccentric phase of 20 cm/s with the dominant leg.

All participants stood an upright position, with the supporting foot (non-dominant leg) placed on top of a box to which a tape measure was attached to measure the distance the subject placed the supporting foot from the holding instrument. This ensured a constant starting position and thus avoided any bias in the execution. Meanwhile, the execution leg (dominant leg) was placed outside of the box, attached to the FEMD with an ankle strap. The subject maintained a comfortable position throughout the execution of the movement, where the knee of the dominant leg was positioned parallel to the holding instrument to sustain balance and simulate the running swing movement.

**Figure 1:**
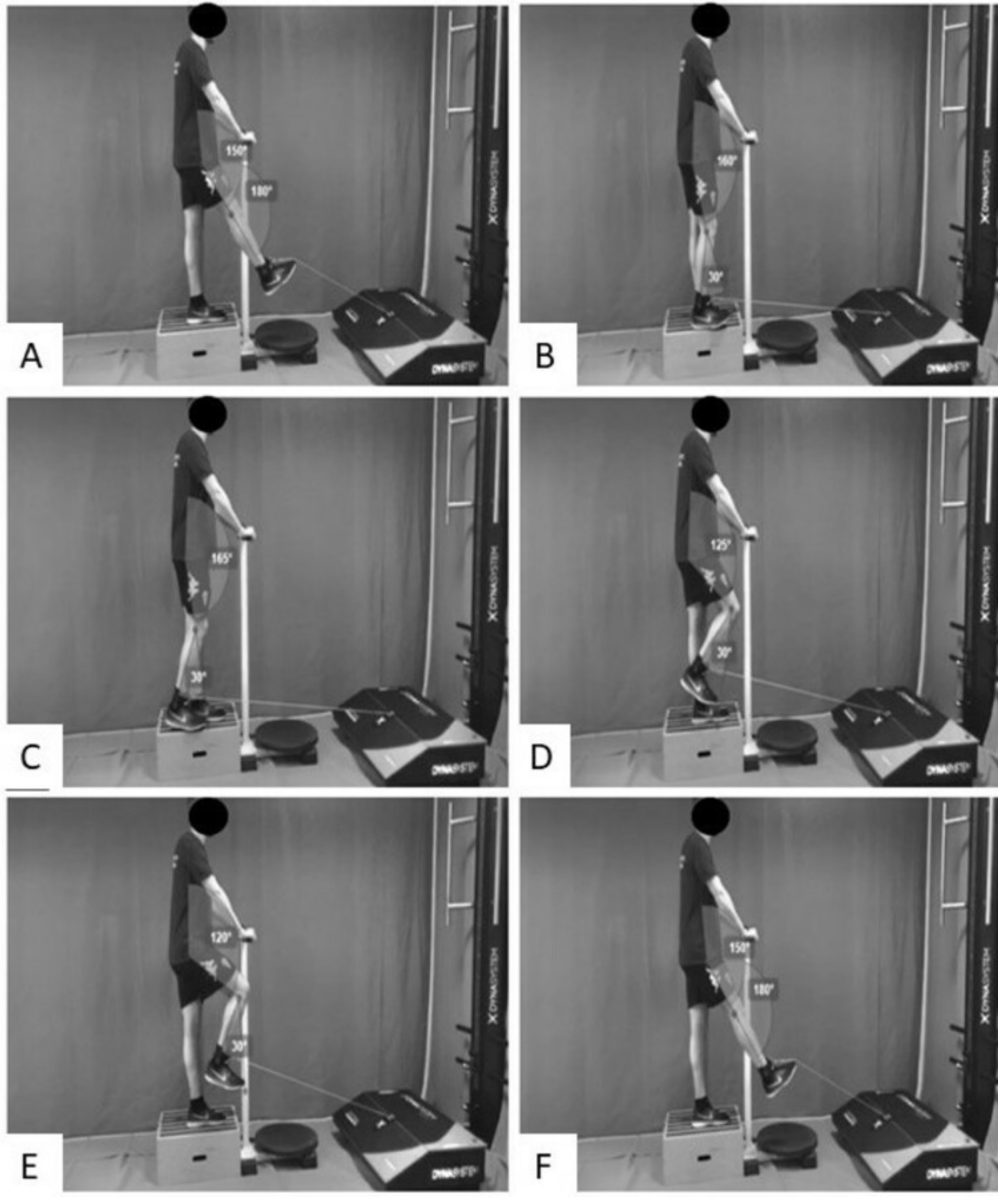
Swing eccentric hamstring exercise protocol. (A) Start position, (B) initial contact stance phase, (C) take off stance phase, (D) initial swing phase, (E) middle-swing phase, (F) terminal-swing phase and start position.

Before the execution of the movement, the leg displacement distance was measured and recorded with the FEMD. The researcher brought the player’s execution leg back fully extended until it was placed parallel to the support leg and a 30° knee flexion was performed (Morin et al., 2015), measured using a goniometer (Fig. 1). After measuring the distance of the supporting foot from the holding instrument and the range of travel of the running leg, the subject performed three series of swing phase of running at three different isokinetic velocities (20–40–60 cm/s) in the eccentric phase, running five repetitions for each velocity. In contrast, the concentric phase remained stable at 20 cm/s (Fig. 1). The order of these different speeds was the same for each trial and participant. To avoid any methodological bias, the subject rested 3 min between each series, thus preventing the appearance of fatigue. The video of the SEHE are available in Supplemental Material.

### Data acquisition and analysis

The average and maximum load (N) of the three series (20–40–60 cm/s) was calculated from the values obtained from the FEMD (Dynasystem^®^, Bangalore).

- Dynasystem^®^: Dynasystem^®^ is a FEMD. Its control core precisely regulates both force and angular velocity through its 2,000 W electric motor. The user is required to apply force on a rope that winds on a roller, thus controlling and measuring both force and linear speed. A load cell senses the tension applied to the rope and the resulting signal goes to an analog-to-digital converter with 12-bit resolution. Displacement and velocity measurements are collected through a 2,500 ppr encoder attached to the roller. Data from the different sensors are collected at a frequency of 1 kHz (Rodriguez-Perea et al., 2021).

### Statistical analysis

The descriptive data are presented as mean ± SD. The normal distribution of the data was confirmed by the Shapiro-Wilk test (*P* > 0.05). Paired sample *t*-test and standardized mean differences (Cohen’s effect size (ES)) were used to compare the magnitude of the load between both testing sessions. The criteria to interpret the magnitude of the ES were as follows: trivial (<0.20), small (0.2–0.59), moderate (0.60–1.19), large (1.20–2.00), and very large (>2.00) (Hopkins et al., 2009). Absolute reliability was assessed by the standard error of measurement (SEM) and coefficient of variation (CV), while relative reliability was assessed by the intraclass correlation coefficient (ICC, model 3.1). Based on a previous study, The criteria for interpreting the magnitude of the ICC were: poor (<0.79), moderate (0.80–0.89), high (>0.90) and CV < 10% was considered reliable (Opar et al., 2013a). For all statistical analysis, the corresponding 95% confidence interval were incorporated into the analysis. Statistical significance was accepted at *p* < 0.05. All reliability assessments were performed by means of a custom spreadsheet (Hopkins, 2017), while other statistical analyses were performed using the software JASP (version 0.14.1).

## Results

In the evaluation of average load during SEHE significant differences between the test-retest were found in the condition of 20 cm/s (*p* = 0.032) and 40 cm/s (*p* = 0.001) with a small effect size (ES = 0.32 and 0.34 respectively). On the other hand, in maximum load there were significant differences in the three conditions between the test-retest (*p* < 0.05), with a small effect size for 20 cm/s (ES = 0.28), 40 cm/s (ES = 0.31) and 60 cm/s (ES = 0.28). Absolute reliability provided stable repeatability for the average and maximum load protocols, with CV less than 10% in all cases (Table 1).

**Table 1:** Test-retest reliability of the FEMD using different isokinetic velocities during SEHE.

	Condition	Session 1^a^ (N)	Session 2^a^ (N)	*p*	F_(1,18)_	ES (95% CI)	CV (95% CI)	SEM (95% CI)	ICC (95% CI)
Average load	20 (cm/s)	336.2 ± 42.3	350.8 ± 47.5	0.032*	5.35	0.32 [−0.58 to 1.23]	5.67 [4.28–8.38]	19.47 [14.71–28.80]	0.83 [0.61–0.93]
	40 (cm/s)	388.2 ± 43.7	402.2 ± 39.3	0.001*	15.05	0.34 [−0.57 to 1.24]	2.80 [2.12–4.14]	11.07 [8.31–6.37]	0.94 [0.84–0.99]
	60 (cm/s)	412.9 ± 58.0	429.3 ± 61.5	0.067	3.77	0.27 [−0.63 to 1.18]	6.17 [4.66–9.13]	25.99 [19.64–38.44]	0.83 [0.61–0.93]
Maximum load	20 (cm/s)	490.2 ± 75.6	510.5 ± 69.3	0.015*	7.19	0.28 [−0.62 to 1.18]	4.66 [3.52–6.89]	23.32 [17.62–34.50]	0.91 [0.78–0.96]
	40 (cm/s)	544.3 ± 87.9	569.8 ± 78.1	0.022*	6.17	0.31 [−0.60 to 1.21]	5.68 [4.29–8.39]	31.62 [23.90–46.77]	0.87 [0.69–0.95]
	60 (cm/s)	597.6 ± 101.3	624.7 ± 92.1	0.030*	5.48	0.28 [−0.62 to 1.18]	5.85 [4.42–8.65]	35.76 [27.02–52.90]	0.88 [0.71–0.95]

**Notes:**

a Values given as mean ± standard deviation.

* *P* < 0.005

*p*, *p*-value; F, F-value; ES, Cohen’s d effect size; CV, coefficient of variation; SEM, standard error of measurement; ICC, intra-class correlation coefficient; 95% CI, 95% confidence interval; N, newton.

The reliability was high for the average load in the condition of 40 cm/s, presenting the highest ICC value (0.94). For maximum load, reliability was high in the condition of 20 cm/s. The manifestation of the most reliable load was the maximum load (ICC = 0.91-0.87).

## Discussion

The main finding of the present study revealed that (I) there was high reliability in the average load for the 40 cm/s isokinetic velocity, with the lowest coefficient of variation (CV = 2.80), (II) reliability was high in the maximum load at 20 cm/s isokinetic velocities in its eccentric phase, with the lowest coefficient of variation (CV = 4.66), and (III) the most absolute reliable strength manifestation was the maximum load (CV range = 4.66–5.85). These results suggest that the FEMD is a reliable device in the evaluation of mean and peak eccentric load during SEHE execution.

Our results are in line with the current review by Dvir & Muller (2019) which states that MIDs can be applied validly and effectively for the assessment and conditioning of specific action muscle patterns, as in this case, SEHE. Research on the use of new technologies for the evaluation of the reliability of functional movements has used iso-inertial devices, such as conical pulley or flywheel, and electric-motor devices, which present lower reliability results than ours for the production of the mean strength and the maximum strength in the eccentric phase during a quarter-squat (ICC = 0.49–0.87; CV = 8–16.6) (Sabido, Hernández-Davó & Pereyra-Gerber, 2018). Similarly, Bollinger et al. (2018) observed high test-retest reliability values for mean strength (*r* = 0.90) and maximum eccentric strength (*r* = 0.92) during the performance of an eccentric hamstring exercise (Romanian deadlift) using a flywheel device. According to Maroto-Izquierdo et al. (2019) although inertial devices produce similar eccentric overload adaptations, electric motor devices have greater potential benefits for eccentric training since they allow for independent modification of concentric and eccentric loads and velocities, a fundamental characteristic of the FEMD used in our study.

After two familiarization sessions, the values obtained (N) in session 2 were slightly higher than those of session 1 for the mean load at 20 and 40 cm/s, and for maximum load at all three isokinetic velocities (20–40–60 cm/s). Although it could be argued that two sessions are sufficient to obtain a reliable measurement, according to Tous-Fajardo et al. (2006) in a study of soccer and rugby players, players who had previous experience in the use of iso-inertial devices showed higher peak eccentric strengths than those who were novices, because some coordination is needed to execute the exercise correctly. These results are in agreement with Sabido, Hernández-Davó & Pereyra-Gerber (2018) which suggest that even in highly trained athletes without eccentric overload training experience, the movement learning period is completed in three sessions. These authors conducted a study comparing the number of familiarization sessions required to obtain a stable and reliable measurement during squat exercise with four different inertial loads (0.025–0.050–0.075–0.100 kg·m^2^), which established the stabilization of data between the 3rd and 4th session (ICC = 0.68–0.87; CV = 8–15.6).

Because the hamstring muscles are more prone to injury during sprints and high-speed actions (Schache et al., 2009; Chumanov, Heiderscheit & Thelen, 2011; Chumanov et al., 2012), it is very interesting to apply functional exercises that are transferable to the actual movement that occurs during the injury mechanism. The SEHE, besides being reliable at all isokinetic velocities (average load and maximum load), complies with the movement pattern that occurs during the sprint. This could result in an improvement in the application of strength in the same direction as they occur during the kick, which could improve the player’s performance in the sprint and strengthen the hamstring injury mechanism (Mendiguchia et al., 2020; Fanchini et al., 2020). Therefore, performing SEHE at high velocities (60 cm/s) could improve the production of maximum eccentric strength from the hamstring muscles, which is considered one of the main risk factors for injury (Lee et al., 2018).

Training programs that increase eccentric strength decrease the number of hamstring injuries and their severity throughout a soccer season (Askling, Karlsson & Thorstensson, 2003; Petersen et al., 2011). NHE is currently the most widely used exercise within these programs, as it reduces the risk of injury by up to 51% (Van Dyk, Behan & Whiteley, 2019) and produces mild to moderate improvements in jumping and sprinting performance (Bautista et al., 2021). In agreement with this author, according to Ishøi et al. (2018) the performance of NHE in amateur players would produce small to medium improvements in sprint performance. Although there is some controversy, as Suarez-Arrones et al. (2021) claim that the improvement in sprint performance is not associated with NHE after the application of a 15–17-week intervention program at the beginning of the season in professional football players. According to Wiesinger et al. (2020) AID and NHE do not reflect the eccentric contraction of the hamstring muscles in the same way since they obviate the influence of the hip joint and lead to divergent estimates of the eccentric strength of the hip joint. Therefore, within the prevention and optimization programs, it would be convenient to introduce multi-articular exercises with a higher level of approximation with respect to the injury mechanism (Suarez-Arrones et al., 2021).

Although the study is current and novel, it has several limitations that should be highlighted. First of all, the sample, being non-professional amateur players, could affect a lower technical and coordination level, which would influence the execution and fluidity of the movement. For this reason, secondly, the study required two sessions of familiarization per player. In fact, some players required three sessions since proper coordination is necessary to execute the gesture correctly. According to these limitations, future research should consider how strength training through the SEHE using a FEMD can influence the transfer of strength applied to actual playing actions in soccer players, as well as its association with injury risk, to implement it within prevention and hamstring optimization programs.

## Conclusions

The main findings of this research show that FEMD is a reliable device to evaluate the eccentric strength of the hamstring muscles through SEHE at the three isokinetic velocities (20–40–60 cm/s) in soccer players showing an acceptable and high reliability. The reliability was high for the average load in the condition of 40 cm/s, presenting the highest ICC value (0.94). For maximum load, reliability was high in the condition of 20 cm/s. The manifestation of the most reliable load was the maximum load (ICC = 0.91–0.87).

## Supplemental Information

10.7717/peerj.11743/supp-1 Supplemental Information 1 Raw Data.

10.7717/peerj.11743/supp-2 Supplemental Information 2 Swing eccentric hamstring exercise.

## Additional Information and Declarations

### Data Availability

The following information was supplied regarding data availability: The raw data showing the test-retest of soccer players evaluated at three different isokinetic velocities are available in the Supplemental File.
